# Monitoring of Follow-Up Reviews in Young Adults After Antidepressant Initiation or Dose Escalation: A Primary Care Audit of NICE Guideline Adherence

**DOI:** 10.1192/bjo.2026.11751

**Published:** 2026-06-30

**Authors:** Varsha Vignesh

**Affiliations:** University of Leicester, Leicester, United Kingdom

## Abstract

**Aims:**

Depression is common amongst young adults and is frequently managed in primary care. Antidepressant medications are commonly initiated or adjusted, however the early stages following this are associated with an increased risk of suicidality in those aged 18-25 years, with the highest risk being observed within the first 28 days of treatment. NICE guidelines therefore recommend close monitoring of this group, including review within one week of initiation or dose increase, followed by further review no later than four weeks. The aim of this audit was to assess compliance with NICE guidelines for follow-up after initiation or dose increase of antidepressant medication in those aged 18-25 years in a primary care setting

**Methods:**

A retrospective audit was conducted in a primary care setting. Electronic medical records were used to identify patients aged 18-25 who were either newly prescribed or had a dose increase of an antidepressant between October 2025 and January 2026. 95 eligible patients were identified, of which 55 were randomly selected. The data collected included the timing and mode of follow-up. The standards that were measured were if patients had been reviewed within one week and within four weeks of antidepressant initiation or dose increase.

**Results:**

3/55 patients (5.5%) were seen within one week and again within four weeks, thus fully compliant with the guidelines. However, only 4/55 patients (7.3%) were reviewed within one week and 8/55 (14.5%) were followed up within four weeks. 35 patients were reviewed during the study period, but not within the recommended guideline timeframes. 12 patients had no documented follow up review since medication initiation. Where reviews occurred, they were most commonly conducted via telephone consultation.

**Conclusion:**

This audit identifies opportunities for improvement in adherence to NICE guidelines for follow-up of antidepressant treatment in young adults within primary care. Given the prevalence of antidepressant prescribing and recognised vulnerability of this age group, untimely follow-up may represent a patient safety concern at a population level. System based interventions to support routine early review may improve adherence, such as automatically booking a 1-week follow-up telephone review at the time of antidepressant initiation or dose increase. Followed by implementing clear pathways for urgent review and escalation if suicidal ideation is identified during follow-up.

